# Symptomatic treatment (using NSAIDS) versus antibiotics in uncomplicated lower urinary tract infection: a meta-analysis and systematic review of randomized controlled trials

**DOI:** 10.1186/s12879-021-06323-0

**Published:** 2021-06-29

**Authors:** Albert Macaire C. Ong Lopez, Charles Jeffrey L. Tan, Antonio S. Yabon, Armin N. Masbang

**Affiliations:** 1 Department of Internal Medicine, St. Luke’s Medical Center-Quezon City, 279 E Rodriguez Sr. Ave, Quezon City, 1112 Metro Manila, Philippines; 2 Department of Internal Medicine, Section of Infectious Disease, St. Luke’s Medical Center-Quezon City, 279 E Rodriguez Sr. Ave, Quezon City, 1112 Metro Manila, Philippines

**Keywords:** Symptomatic treatment, NSAIDS, Non-steroidal anti-inflammatory drugs, Antibiotics, Uncomplicated UTI

## Abstract

**Background:**

Current guidelines recommend empiric antibiotics as first-line treatment for uncomplicated UTI. Despite proven benefits in treatment, antibiotic resistance rates remain on the rise. This meta-analysis aims to determine whether non-steroidal anti-inflammatory drugs can serve as an effective and safe option in the treatment of uncomplicated lower UTI among non-pregnant women compared to antibiotics.

**Methods:**

A systematic literature search in PUBMED, CENTRAL, and ACP databases from inception to April 2021 was conducted to identify randomized controlled trials that compare the use of non-steroidal anti-inflammatory drugs versus antibiotics in non-pregnant women ≥18 years old with uncomplicated lower urinary tract infection. Primary outcomes were symptom resolution of UTI by Day 3 or 4 of intervention, and upper UTI complications. Secondary outcomes include persistence of positive urine culture despite treatment and need for another rescue antibiotic. Random and fixed-effects model for dichotomous data using Mantel-Haenszel and Peto odds method were reported at 95% CI followed by sensitivity analysis for substantial heterogeneity.

**Results:**

Four RCTs involving 1165 patients were analyzed. The probability of having a symptom resolution by Day 3 or 4 with NSAID use is only less than three-fourths of that with antibiotic treatment (RR: 0.69, 95% CIs [0.55, 0.86], *p* = 0.0008, I^2^ = 73%, moderate certainty of evidence). The odds of developing upper UTI complications with use of NSAIDs are 6.49 to 1 for antibiotics (Peto OR: 6.49, 95% CIs [3.02, 13.92], *p* < 0.00001, I^2^ = 0%, moderate certainty of evidence). Secondary analysis showed that the NSAID group is 2.77x more likely to have persistence of a positive microbiologic urine culture than the antibiotic group (RR: 2.77, 95% CIs [1.95, 3.94], *p* < 0.00001, I^2^ = 36%, moderate certainty of evidence). Treatment with NSAIDs are three times more likely to use a secondary or rescue antibiotic due to persistent or worsening symptoms as compared to antibiotics (RR: 3.16, 95% CIs [2.24, 4.44], *p* < 0.00001, I^2^ = 47%, low certainty of evidence).

**Conclusion:**

Antibiotic treatment was more effective than use of non-steroidal anti-inflammatory drugs for acute uncomplicated lower urinary tract infection with an overall moderate certainty of evidence.

**Supplementary Information:**

The online version contains supplementary material available at 10.1186/s12879-021-06323-0.

## Background

Urinary tract infection remains as one of the leading causes of morbidity worldwide, with an estimated prevalence of 0.7% [1]. An estimated 50–60% of women report having UTI in their lifetime [2]. In the United States alone, UTIs account for nearly 7 million office visits, 1 million emergency department visits, and more than 100,000 hospitalizations with an annual healthcare expenditure cost of at least 1.6 billion dollars [3].

Clinically, UTIs can be categorized as either uncomplicated or complicated. Uncomplicated UTI is further differentiated into lower UTI or cystitis, and upper UTI or pyelonephritis. Uncomplicated lower UTI is suspected in mostly premenopausal, non-pregnant women presenting with acute symptoms of dysuria, urgency, frequency, lower abdominal pain and absence of fever without significant structural or functional abnormalities within the urinary tract.

Current primary care guidelines recommend empiric antibiotics as the first line of treatment for uncomplicated urinary tract infection [4]. A meta-analysis of randomized controlled trials showed superiority of antibiotic therapy over placebo in treatment of adult non-pregnant women with uncomplicated cystitis (OR = 4.67, 95% CIs [2.34, 9.35], I^2^ = 58%) [5].

Despite the benefits of antibiotic treatment in achieving symptomatic and bacteriologic cure, antibiotic resistance rates are on the rise. The most common pathogen is uropathogenic *E. coli* (UPEC). In a recent study done in Southern China primary care, antibiotic prescription rate is at 82.2%. Isolates of *E.coli* revealed resistance rates to ampicillin, co-trimoxazole, ciprofloxacin, amoxicillin and nitrofurantoin at 59.8, 31.8, 23.4, 1.9 and 0.9% respectively [6]. In addition, antibiotic adverse event resulting in health service use and cost can’t be undermined. Oral sulfonamides (23.2%; 20.6–25.8%), penicillin (20.8%; 19.3–22.4%), and quinolones (15.7%; 14.2–17.1%) were the 3 most common antibiotic classes that are implicated in emergency visits due to antibiotic associated adverse effects. Rashes and pruritus were the commonly documented adverse reactions across all antibiotics and majority is due to sulfonamides [7].

With the advent of antimicrobial stewardship, combating the overuse of antibiotics and dealing with increasing resistance rates, it is essential to determine if there are other efficacious alternative options to antibiotics in the treatment of uncomplicated lower urinary tract infections.

One of the strategies is to delay institution of antibiotics. In the study by Little et al., there were no significant differences in the severity of symptoms for 2 to 4 days after seeing a health professional between immediate vs delayed antibiotics (at least 48 h) (mean frequency of symptoms on a 0 to 6 scale: immediate antibiotics 2.15, delayed antibiotics 2.11; *p* = 0.177). However, patients who delayed starting antibiotics for at least 48 h had symptoms of 37% longer duration than those taking immediate antibiotics (incidence rate ratio 1.37, 95% CIs [1.11,1.68], *p* = 0.003) [8].

Another alternative strategy is symptomatic treatment with non-steroidal anti-inflammatory drug. This is based on studies which suggest uncomplicated urinary tract infection is a self-limiting condition, with a favorable natural course of the disease [9–11]. Several recent randomized controlled trials have compared symptomatic treatment using non-steroidal anti-inflammatory drugs to antibiotics in the treatment of uncomplicated UTI. A 2010 landmark pilot randomized controlled trial by *Bleidorn et.al.* with 79 participants revealed that on Day 4 of illness, the ibuprofen group reported fewer symptoms than the ciprofloxacin group (symptom resolution 58.3% vs 51.5%; *p* = 0.744), which then supported the assumption of non-inferiority of NSAIDS compared to antibiotics in the treatment of symptomatic uncomplicated UTI [12]. Three other randomized controlled studies followed, with conflicting results [13–15].

In this paper, we performed a meta-analysis to assess whether the use of non-steroidal anti-inflammatory drugs can serve as an effective and safe alternative option in the treatment of uncomplicated lower urinary tract infection among non-pregnant women > 18 years old as compared to antibiotics.

### Research question

Among non-pregnant women with symptoms of uncomplicated lower urinary tract infection, how effective and safe is treatment with a non-steroidal anti-inflammatory drug (NSAIDs) as compared to antibiotic monotherapy using a meta-analysis of randomized controlled trials?

### Specific objectives

The general objective of this meta-analysis is to determine the efficacy of using a non-steroidal anti-inflammatory drug as compared to antibiotic therapy in achieving symptomatic cure for uncomplicated lower urinary tract infection in non-pregnant women. Our specific objectives include assessment of complications of non-steroidal anti-inflammatory drugs as compared to antibiotics on a) upper UTI infection which comprised of febrile UTI and pyelonephritis, b) post-treatment follow-up urine microbiologic cultures, and c) use of secondary antibiotics due to persistent or worsening symptoms of UTI.

## Methods

### Criteria for considering studies for this review

Studies were selected on the basis of a randomized-controlled study design, regardless of blinding status, or a specific study design such as cluster-randomized or cross-over type. Pre-specified inclusion criteria for type of participants comprised of non-pregnant women aged > 18 yrs. old with one or more signs or symptoms of acute uncomplicated lower urinary tract infection: urinary frequency, dysuria, urgency, hematuria or suprapubic pain. Only included in this review are those randomized controlled trials that compare use of non-steroidal anti-inflammatory drugs versus antibiotics as active control. The type of non-steroidal anti-inflammatory agent or antibiotic drug, dosage/intensity, frequency and duration were not restricted in our inclusion criteria.

Primary outcomes for inclusion were 1) symptom resolution by Day 3 or 4 of treatment as defined by the respective studies, and 2) complications of primary treatment defined as progression to upper urinary tract infection subdivided as a) acute pyelonephritis presenting as fever with flank pain or costovertebral tenderness and urinary symptoms of dysuria, frequency, urgency, hematuria or suprapubic pain, b) febrile UTI manifesting as fever with urinary symptoms of frequency, dysuria, urgency, hematuria or suprapubic pain but without back pain, flank pain or CVA tenderness.

Secondary outcomes were patients with 1) persistence of a positive urine culture defined as growth of bacterial isolate on culture media after a maximum of 4 days of primary treatment and b) secondary treatment with new antibiotics defined as use of a rescue antibiotic after primary treatment with non-steroidal anti-inflammatory drugs or the need to switch to another antibiotic class group from an initial different antibiotic therapy due to persistent or worsening symptoms of UTI.

This study excluded non-randomized trials comprising cross-sectional studies, case-control reports, and quasi-experimental study designs. Participants that encompass pregnant women, the immunocompromised, recurrent urinary tract infection, and with an initial diagnosis of pyelonephritis or an upper urinary tract infection were also not included.

### Information sources and search strategies for identification of studies

This meta-analysis was performed in accordance to the PRISMA (Preferred Reporting Items for Systematic Reviews and Meta-analyses) 2020 statement [16]. Three independent reviewers (A.M.C.-O.L., C.L.-T., and A.S.-Y.) performed a systematic search and evaluation of randomized-controlled trials on non-steroidal anti-inflammatory treatment for uncomplicated urinary tract infection published from inception up to April 2021, in the following scientific search engines: PUBMED, Cochrane Central Register of Controlled Trials, and ACP Journal Club. The search strategy used was “(non-steroidal anti-inflammatory drugs OR ibuprofen OR diclofenac) AND (cystitis OR urinary tract infection OR uncomplicated urinary tract infection)”. Unpublished trials and ongoing studies in national and international trial registers (ClinicalTrials.gov, ISRCTN Register, EU Clinical Trials Register and WHO ICTRP), dissertation and thesis databases, conference abstracts and other grey literature sources were also sought in the relevant search. The comprehensive search was not restricted by any language or publication date filter.

### Selection of studies

Three authors (A.M.C.-O.L., C.L.-T., and A.S.-Y.) independently performed a systematic process for selecting studies for inclusion in the review. After identifying all the studies through database searching, any duplicate records of the same report were removed. Manuscript titles and abstracts were examined carefully and included only those that met the criteria for this review. Subsequently, the reviewers independently retrieved the full text of the potentially relevant reports. The full text reports were then scrutinized for eligibility and excluded those that did not conform with the inclusion and exclusion criteria. If there were missing data results or inconsistencies, the reviewers attempted to correspond with the original investigators. Any clarification or discrepancies in the study selection process were resolved accordingly by the authors via a consensus and consultation with a fourth expert investigator (A.N.-M.).

### Data collection

Review authors have planned in advance the relevant data to be collected in this meta-analysis and systematic review. A data collection form was created which included the citation details, study design, total study duration, type and number of participants, study location, study inclusion and exclusion criteria, baseline patient characteristics, description of the intervention and control, relevant outcome of interest, and results. This data collection form guaranteed some consistency in the process of data abstraction, and deemed necessary in comparing data.

### Data extraction and management

Four data extractors, an infectious disease specialist, and three of the review authors (A.M.C.-O.L., C.L.-T., and A.S.-Y.) independently extracted data from every report using a data collection form created by the authors. Any disagreements were resolved by reviewing again the data, and through consensus.

### Assessment of risk of bias in included studies

Three independent reviewers (A.M.C.-O.L., C.L.-T., and A.S.-Y.) critically appraised each trial using the Cochrane Risk of Bias (RoB 2.0) tool. The domains in RoB 2.0 include bias arising from the randomization process; bias due to deviations from intended interventions; bias due to missing outcome data; bias in measurement of the outcome; and bias in selection of the reported result. Judgement of overall risk-of-bias was subdivided into “low risk of bias”, “some concerns” or “high risk of bias.” Any discrepancies in risk assessment were settled through constructive discussions between authors.

### Data synthesis and analysis

Data synthesis and analysis were performed using Revman 5.4.1 for Windows 10. The effect measure of choice for the majority of each outcome was reported as risk ratio for dichotomous data reported at 95% confidence interval. However, in dichotomous outcomes with zero-event rates, the Peto odds ratio was then reported. A 2-sided *p* < 0.05 was determined as statistically significant.

Statistical heterogeneity between randomized controlled trials were assessed using the I^2^ statistics, with an interpretation of I^2^ value of 30–60% representing moderate heterogeneity, 50–90% indicating substantial heterogeneity, and 75–100% interpreted as considerable heterogeneity. If heterogeneity was identified, the random-effects analysis model was used; otherwise, the fixed-effect estimates were reported. The Mantel-Haenszel method was used primarily in the analysis in consideration for some studies with small sample sizes and lower event rates. On the other hand, the Peto’s method was performed for dichotomous outcome data with zero cell counts and the studies having relatively similar numbers in experimental and comparator groups. Assessment of publication or reporting bias was evaluated using funnel plot.

When significant heterogeneity was still detected even after exploring the use of random-effects model, a sensitivity analysis was performed by doing a systematic approach of repeating the initial analysis through exclusion of studies that are deemed not eligible on the basis of sample size, methodological quality, variances on patient population, intervention features and data. A *L’Abbé* plot was likewise used for investigating the potential sources of heterogeneity. Sources of heterogeneity were identified and discussed in this review.

### Certainty assessment

Three independent reviewers (A.M.C.-O.L., C.L.-T., and A.S.-Y.) evaluated the certainty of evidence using the The Grades of Recommendation, Assessment, Development and Evaluation Working Group (GRADE) approach. Rating criteria for considering lowering or raising the certainty of evidence was dependent on these factors which consist of risk of bias, inconsistency, indirectness, imprecision, publication bias, large effect, dose response and other plausible confounding bias.

## Results

### Study selection

**Fig. 1:**
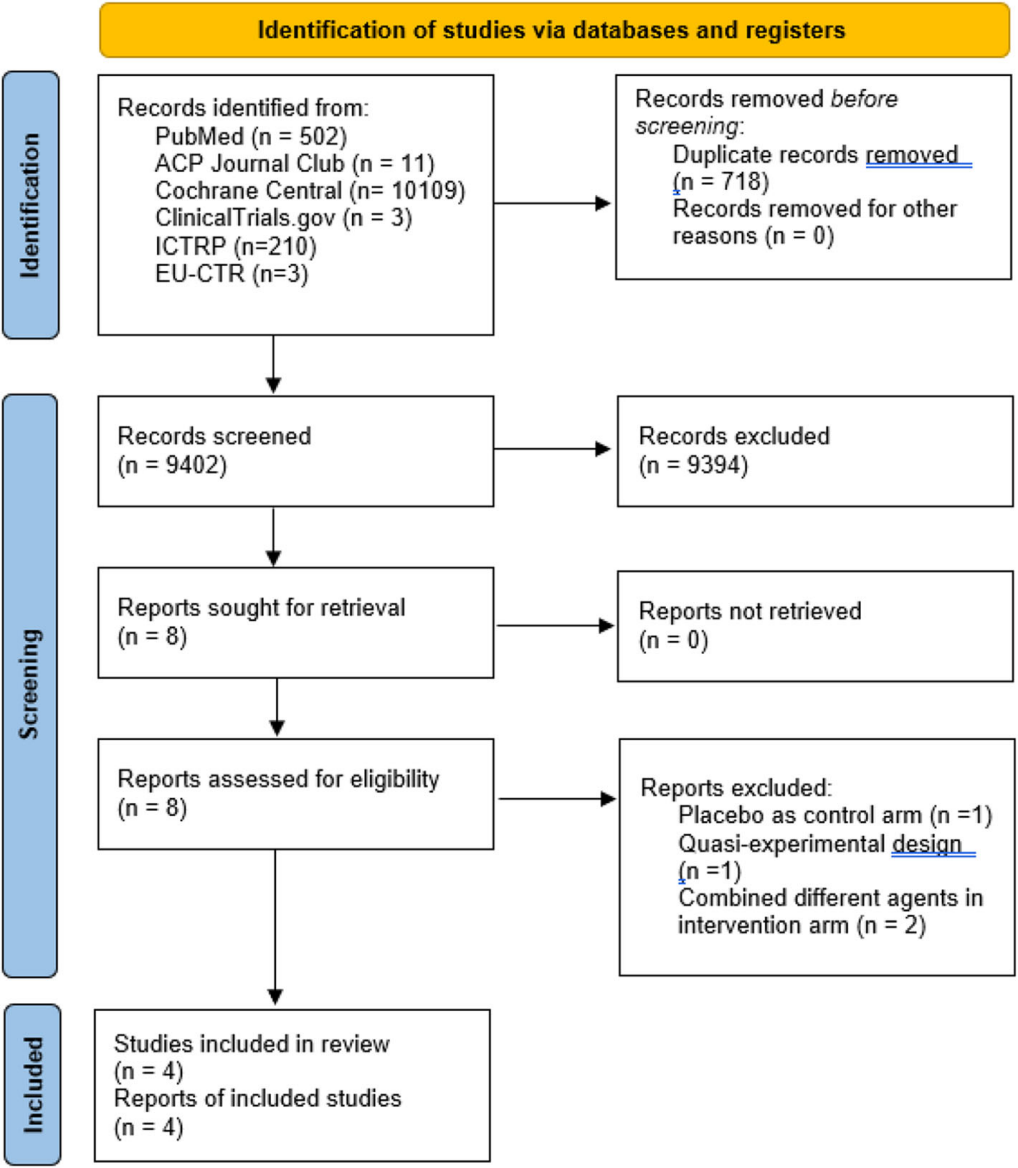
PRISMA 2020 flow diagram for new systematic reviews

Out of the 10,838 records that were identified through database searching, 718 duplicate records were removed, and 9394 articles were excluded after screening the titles and/or abstracts. 8 full-text articles were assessed for eligibility with final exclusion of 4 articles. *Moore* et al. [17] used *uva-ursa* extract and/or ibuprofen for symptomatic treatment of uncomplicated acute urinary tract infection but a comparison was not made with antibiotics for the control arm. *Aloush* et al [18] used a prospective quasi-experimental design, thus the study was excluded. *Jamil* et al [19] compared both potassium citrate and flurbiprofen with ciprofloxacin monotherapy. Similarly, *Ko et. al* [20] compared both the efficacy of aceclofenac in combination with cepodoxime to single-agent cepodoxime for symptom resolution in patients with acute uncomplicated cystitis. The use of another medication on top of the non-steroidal anti-inflammatory drug in the intervention group may substantially affect the heterogeneity in the analysis, therefore, both studies were excluded. After detailed evaluation, a total of four randomized controlled trials were included in this review (Fig. 1) [12–15]. The trials were specifically designed to investigate the use of non-steroidal anti-inflammatory drugs versus antibiotic therapy in uncomplicated urinary tract infection. No ongoing similar studies were identified in the relevant search.

### Study characteristics

Across 4 trials, a total of 1165 participants were included, with 584 in the NSAID group and 560 in the antibiotic group. There were no major differences in the baseline characteristics between both groups in all the individual trials. The mean age of participants in the non-steroidal anti-inflammatory drug group ranged from 28.1 to 44.6 years, while in the antibiotic group, it ranged from 28.5 to 43.7 years. All participants in the study were non-pregnant women. Ibuprofen and diclofenac were the non-steroidal anti-inflammatory agents studied for the experimental group, whereas ciprofloxacin, fosfomycin, norfloxacin and pivmecillinam were the antibiotics used for the control group. The dosages of the different medications varied across studies. The course of treatment was 3 days for all the included trials. All individual trials reported outcomes of urinary tract symptoms after treatment with non-steroidal anti-inflammatory drug versus antibiotic as symptom burden and/or resolution. Three trials reported on resolution of UTI symptoms at day 4 with day of randomization defined as day 0 [12, 13, 15], while only one trial reported symptom resolution on day 3, with its day of randomization defined as day 0 [14]. Three studies evaluated on the after-treatment complication rates of upper UTI described as febrile UTI and/or pyelonephritis. Three trials reported on the presence of positive urine cultures on follow-up with one study on Day 7, 10 and 14 respectively. All trials did report on the frequency of secondary antibiotic treatment for persistent UTI symptoms. Table 1 summarizes the characteristics of the included studies.

**Table 1:** Study characteristics of NSAID versus Antibiotic use in Uncomplicated UTI

First author, year	Study design, location	Study population	Inclusion criteria	Exclusion criteria	NSAID vs Antibiotic	Outcomes
Bleidorn, 2010	Multicenter, double-blinded, RCT, pilot trial, Germany	Non-pregnant women aged 18–85 y/o, with uncomplicated UTI	At least one of the main UTI symptoms dysuria and frequency	Signs of upper UTI symptoms (fever, back pain) Pregnancy Comorbidities: DM, CKD GIT abnormalities or past urinary surgery, Urine catheterization, immunosuppressive therapy UTI within the last two weeks Current use of antibiotics or NSAIDs; History of GI ulcers Epilepsy Allergies	Ibuprofen vs Ciprofloxacin	Primary: Symptom resolution on Day 4 Secondary: Burden of symptoms on Day 4 and, symptom resolution on Day 7 and frequency of relapses until Day 28, and incidence of adverse events
Gagyor, 2015	Multicenter, double-blinded, RCT, Germany	Non-pregnant women aged 18–65 y/o, with uncomplicated UTI	Dysuria and/or frequency/urgency of micturition, with or without lower abdominal pain	Any signs of upper UTI (fever, loin tenderness); Pregnancy Renal diseases UTI within the past two weeks Urinary catheterization. Recent NSAID or antibiotics use History of GI ulcers or severe acute or exacerbated chronic conditions	Ibuprofen vs Fosfomycin	Primary: Number of all courses of antibiotic treatment on Day 0–28, Burden of symptoms on Day 0 to 7 Secondary: Number of severe adverse events, complications, relapses up to Day 28, and within 6 and 12 months, women without symptoms at day 4 and 7, symptom load until Day 4, activity impairment on Day 1–7
Kronenberg, 2017	Multicenter, double-blinded, RCT, Switzerland	Non-pregnant women aged 18–70 y/o, with uncomplicated UTI	One or more symptoms or signs typical of acute lower UTI (dysuria, frequency, macrohematuria, cloudy or smelly urine) or self-diagnosed symptomatic cystitis (urine dipstick was positive for nitrite or leucocytes, or both)	Pregnant women and women Signs of upper UTI: (fever, costovertebral pain or tenderness, rigors, and nausea or vomiting) GIT abnormalities Comorbidities (DM, GI ulcer, IBD, liver cirrhosis, CKD, CHF) Psychiatric illness or dementia Documented immunosuppression Hypersensitivity reactions Women with vaginal symptoms (discharge, irritation) Bladder catheterization Recurrent UTI Antibiotic treatment during the past four weeks UTI symptoms > 7 days	Diclofenac vs Norfloxacin	Primary: Resolution of symptoms at day 3 Secondary: Use of any antibiotic up to Day 30, resolution of symptoms on Day 7, complete absence of symptoms on Days 3 and 7, use of rescue antibiotic up to Day 3, negative urine culture result on Day 10, reconsultations because of UTI up to day 30, adverse events, serious adverse events, European quality of life.
Vik, 2018	Multicenter, double-blinded, noninferiority, RCT, Norway, Denmark, Sweden	Non-pregnant women aged 18–60 y/o, with uncomplicated UTI	Dysuria combined with either increased urinary frequency or urinary urgency or both, with or without visible hematuria	Signs of upper UTI (fever, upper back pain) UTI symptoms for > 7 days Allergies/adverse reactions to penicillin or ibuprofen Breastfeeding a child under 1 month of age Vaginal irritation/discharge Comorbids: diabetes; kidney disease; genetic aciduria; severe gastritis; ulcerative colitis; Crohn’s disease; low platelets); Immunosuppressive therapy, or blood-thinning drugs Previous pyelonephritis Urinary catheterization Symptoms of a UTI within the last 4 weeks Antibiotic use within the last 2 weeks	Ibuprofen vs Pivmecillinam	Primary: Proportion of patients who felt cured by Day 4 Secondary: Proportion of patients in need of secondary treatment with antibiotics, proportion of patient with positive second urine culture, in need of a medical consultation within 4 weeks of follow-up, cases of pyelonephritis

**Table 2:** GRADE Summary of Findings

Non-steroidal anti-inflammatory drugs compared to antibiotics for uncomplicated acute urinary tract infection												
**Patients or population**: Non-pregnant women aged > 18 yrs. old with acute uncomplicated lower urinary tract infection												
**Settings**: Community-based												
**Intervention:** Non-steroidal anti-inflammatory drugs												
**Comparison:** Antibiotics												
**Certainty assessment**							**№ of patients**		**Effect**		**Certainty**	**Importance**
**№ of studies**	**Study design**	**Risk of bias**	**Inconsistency**	**Indirectness**	**Imprecision**	**Other considerations**	**non-steroidal anti-inflammatory drugs**	**antibiotics**	**Relative** **(95% CI)**	**Absolute** **(95% CI)**		
Symptom resolution by Day 3 or 4												
1144	randomised trials	not serious	serious	not serious	not serious	none	254/584 (43.5%)	373/560 (66.6%)	**RR 0.69** (0.55 to 0.86)	**206 fewer per 1000** (from 300 fewer to 93 fewer)	⨁⨁⨁◯ MODERATE	CRITICAL
Complication of upper urinary tract infection												
1096	randomised trials	serious	not serious	not serious	not serious	none	26/555 (4.7%)	1/541 (0.2%)	**OR 6.49** (3.02 to 13.92)	**10 more per 1000** (from 4 more to 23 more)	⨁⨁⨁◯ MODERATE	CRITICAL
Positive urine culture post-treatment												
681	randomised trials	serious	not serious	not serious	not serious	none	100/350 (28.6%)	34/331 (10.3%)	**RR 2.77** (1.95 to 3.94)	**182 more per 1000** (from 98 more to 302 more)	⨁⨁⨁◯ MODERATE	IMPORTANT
Use of rescue or secondary antibiotic												
1165	randomised trials	serious	serious	not serious	not serious	none	243/591 (41.1%)	72/574 (12.5%)	**RR 3.16** (2.24 to 4.44)	**271 more per 1000** (from 156 more to 431 more)	⨁⨁◯◯ LOW	IMPORTANT

***CI*** Confidence interval; ***RR*** Risk ratio; ***OR*** Odds ratio

### Risk of bias in included studies

**Fig. 2:**
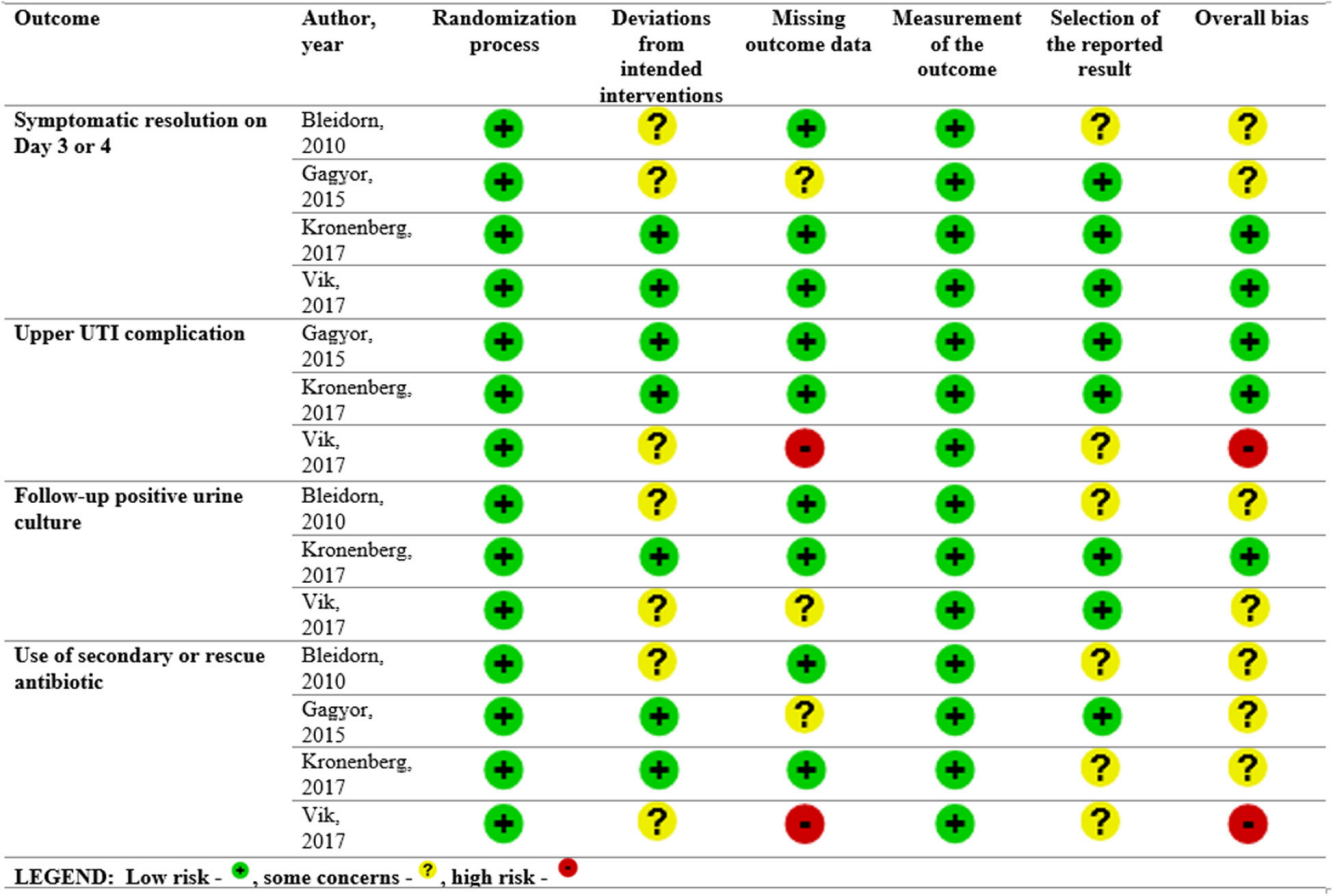
Cochrane risk of bias 2.0 assessment

Risk of bias assessment of individual trials were summarized in Fig. 2. For the outcome symptom resolution by Day 3 or 4 of treatment, two trials had low risk of bias, while the other two trials presented some concerns. For upper urinary tract infection complication, one trial had high risk of bias. For positive urine culture on follow-up, two trials had some concerns, with the remaining one had low risk of bias. For the outcome use of secondary or rescue antibiotics, one trial was judged to be of high risk of bias, while three studies presented some concerns.

Overall, all trials employed a computer-generated randomization sequence in varying block sizes. In each individual trial, allocation concealment was adequate prior to assignment. All studies performed a double-blinded study design. The study participants, treating doctor, study nurses and outcome assessors were blinded from knowledge of which intervention the participant received. Some biases that were identified in the critical appraisal include possible use of inappropriate analysis (per-protocol instead of intention-to-treat) to estimate the effect of assignment to intervention, presence of missing outcome data that could depend on its true value, and data that were not analyzed in accordance with a pre-specified analysis plan. A more detailed assessment of risk of bias of the included studies is accessible in the online supplementary material.

### Outcomes of the meta-analysis

**Fig. 3:**
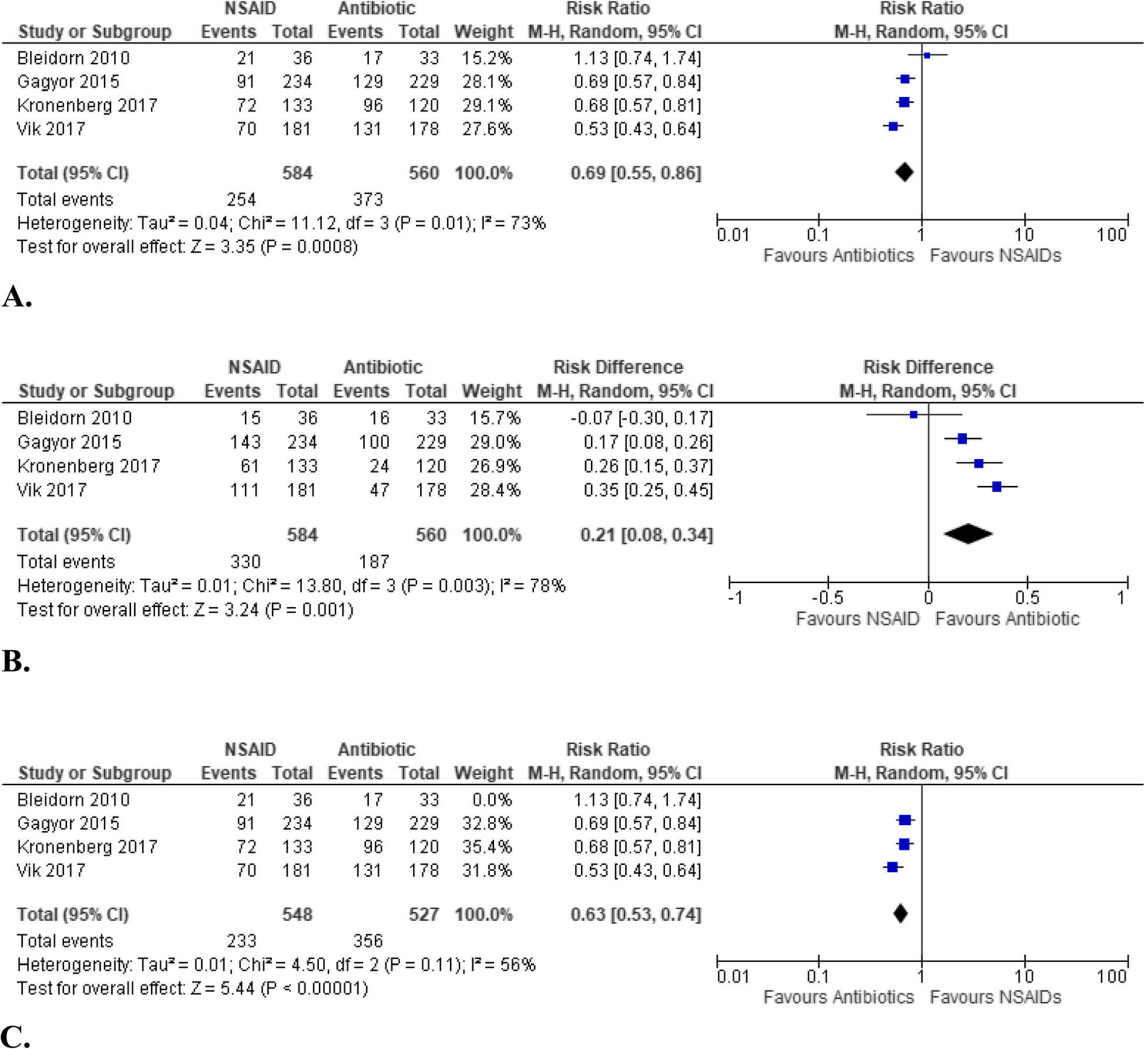
**A** Forest plot of comparison: Summary of symptomatic cure on Day 3 or 4 of treatment between NSAID and Antibiotic use in women with uncomplicated UTI. **B**. Forest plot of comparison: Risk difference between NSAID and Antibiotic use on persistence of symptoms on Day 3 or 4 of treatment. **C**. Forest plot of comparison: Sensitivity analysis on Day 3 or 4 of treatment between NSAID and Antibiotic use in women with uncomplicated UTI

**Fig. 4:**
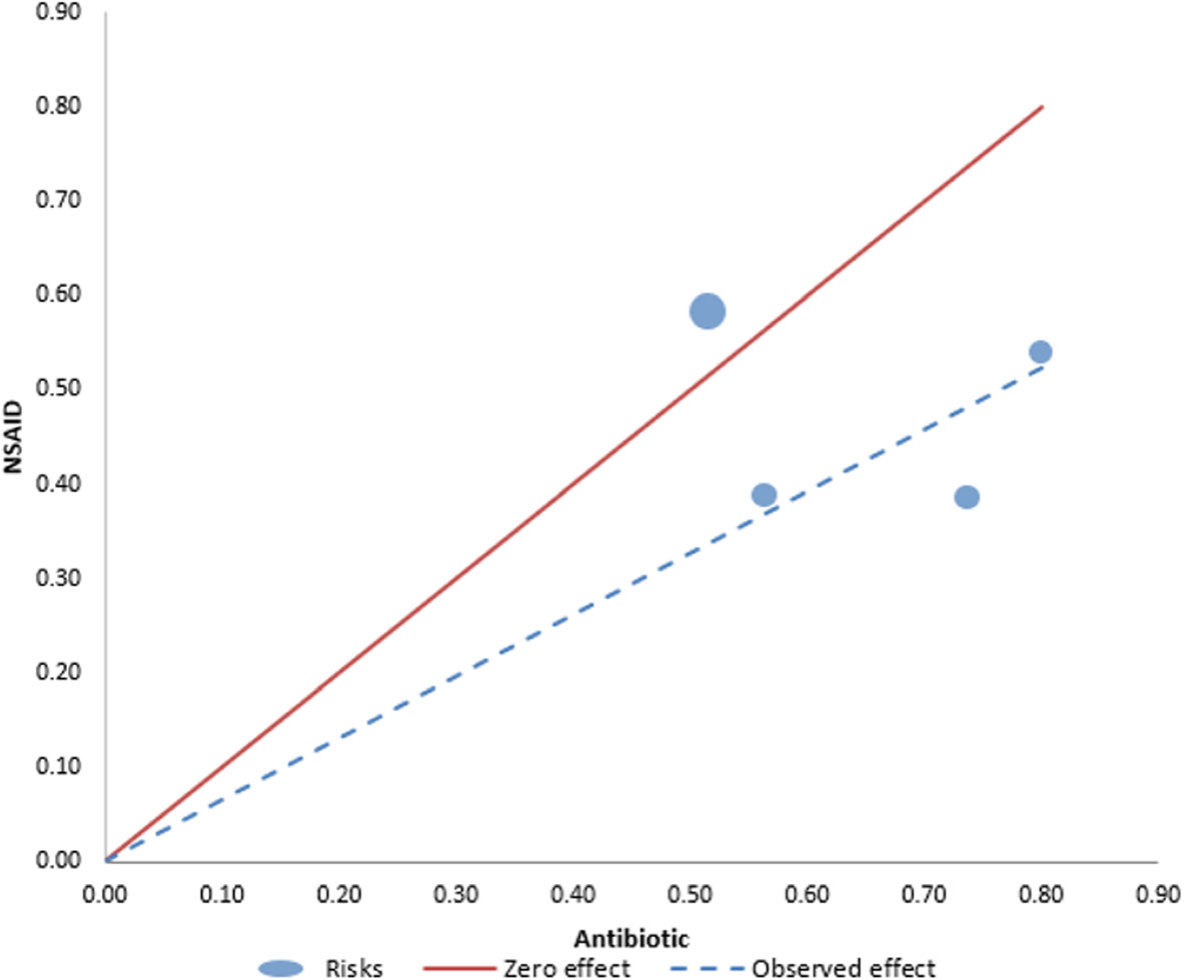
L’Abbe plot: Symptom resolution response rates to treatment vs control

The probability of having a symptom resolution by Day 3 or 4 with NSAID use is only less than three-fourths of that with antibiotic treatment (RR: 0.69, 95% CIs [0.55, 0.86], *p* = 0.0008, I^2^ = 73%, moderate certainty of evidence) (Fig. 3A). Between-trial heterogeneity was identified in this analysis with an I^2^ of 73% and as reflected by the *L’Abbé* plot (Fig. 4). The dashed line runs through the bottom-right sector of the *L’Abbé* plot signifying that the pooled effect estimate favors the antibiotics group in terms of symptom resolution by Day 3 or 4. The pilot study by *Bleidorn et. al*. [12] lies above the line of equality, while most studies fall near the dashed line. This suggests that the *Bleidorn et. al.* study may have contributed to the between-study heterogeneity, which distorted the overall pooled effect. A sensitivity analysis was subsequently performed, which showed a decrease in I^2^ from 73 to 56% (Fig. 3C) after the exclusion of the pilot 2010 study by *Bleidorn et. al* [12], but still with moderate heterogeneity. The major source of heterogeneity identified in this analysis is the difference in their methodology on the definition of symptom resolution. The *Bleidorn et. al.* and *Gágyor et. al.* study group defined symptom resolution as the number of patients with a symptom sum score of 0 (dysuria, frequency/urgency, and lower abdominal pain). Each symptom was graded on a scale from 0 (none) to 4 (very strong). *Kronenberg et. al.* used a questionnaire with five severity UTI symptoms (dysuria, frequency, urgency, abdominal pain, pain in lower back) categorized on a scale from 0 to 6. Symptom resolution was defined on the basis of 2 or less points. On the other hand, *Vik et. al.* defined symptom resolution as the proportion of patients who felt cured on Day 4 with a symptom sum score of 0 using a patient diary. Its sum score consisted of dysuria, urinary frequency, and urgency graded on a scale from 0 (normal) to 6 (as bad as it could be). Other sources of heterogeneity include use of non-validated questionnaire as stated by the respectives studies, and differences in baseline symptom sum scores which could underestimate or overestimate the results.

**Fig. 5:**
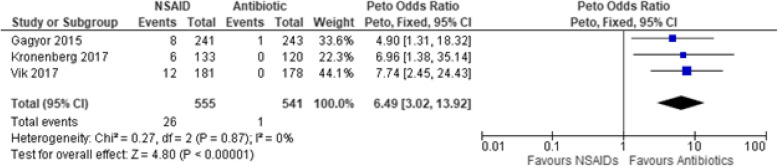
Forest plot of comparison: Summary of complications [Pyelonephritis and Febrile UTI] in women with uncomplicated UTI randomized to either NSAID or Antibiotic

**Fig. 6:**
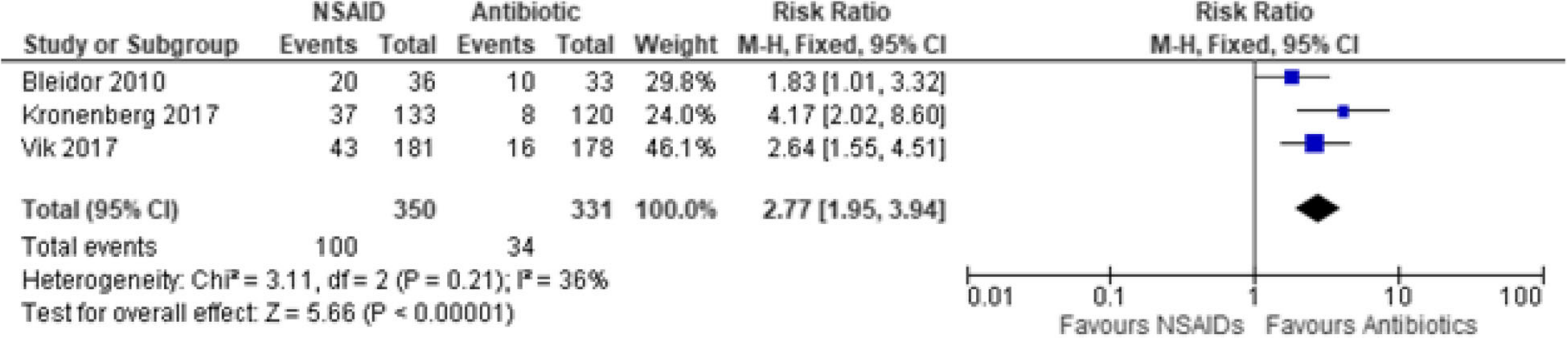
Forest plot of comparison: Summary of follow-up positive urine cultures *[Bleidorn on day 7, Kronenberg on day 10, Vik on day 14]* in women with uncomplicated UTI randomized to either NSAID or Antibiotic

**Fig. 7:**
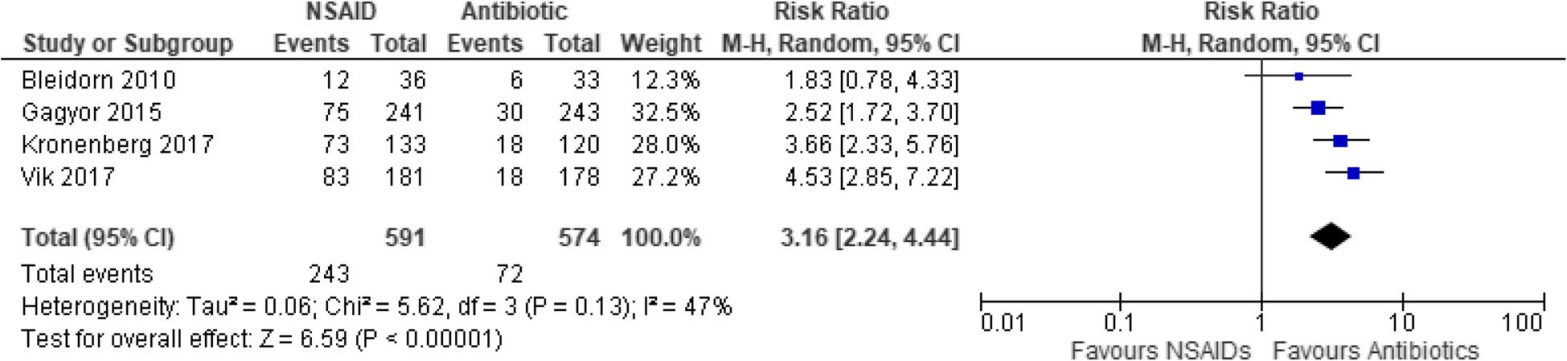
Forest plot of comparison: Summary of use of secondary antibiotic for persistent or worsening UTI symptoms *[Bleidorn by day 7, Gágyor by day 28, Kronenberg by day 30, Vik by day 28]* in women with uncomplicated UTI randomized to either NSAID or Antibiotic

Another primary outcome in this study showed that the odds of developing upper UTI complications (comprising of pyelonephritis and febrile UTI) with the use of NSAIDs are 6.49 to 1 for antibiotics (Peto OR: 6.49, 95% CIs [3.02, 13.92], *p* < 0.00001, I^2^ = 0%, moderate certainty of evidence) (Fig. 5). Secondary outcome analysis showed that the NSAID group is 2.77 times more likely to have persistence of a positive microbiologic urine culture than the antibiotic group even after the initial treatment (RR: 2.77, 95% CIs [1.95, 3.94], *p* < 0.00001, I^2^ = 36%, moderate certainty of evidence) (Fig. 6). Moreover, treatment with NSAIDs are three times more likely of having a secondary or rescue antibiotic due to persistent or worsening symptoms as compared to antibiotics (RR: 3.16, 95% CIs [2.24, 4.44], *p* < 0.00001, I^2^ = 47%, low certainty of evidence) (Fig. 7).

### Reporting Bias

**Fig. 8:**
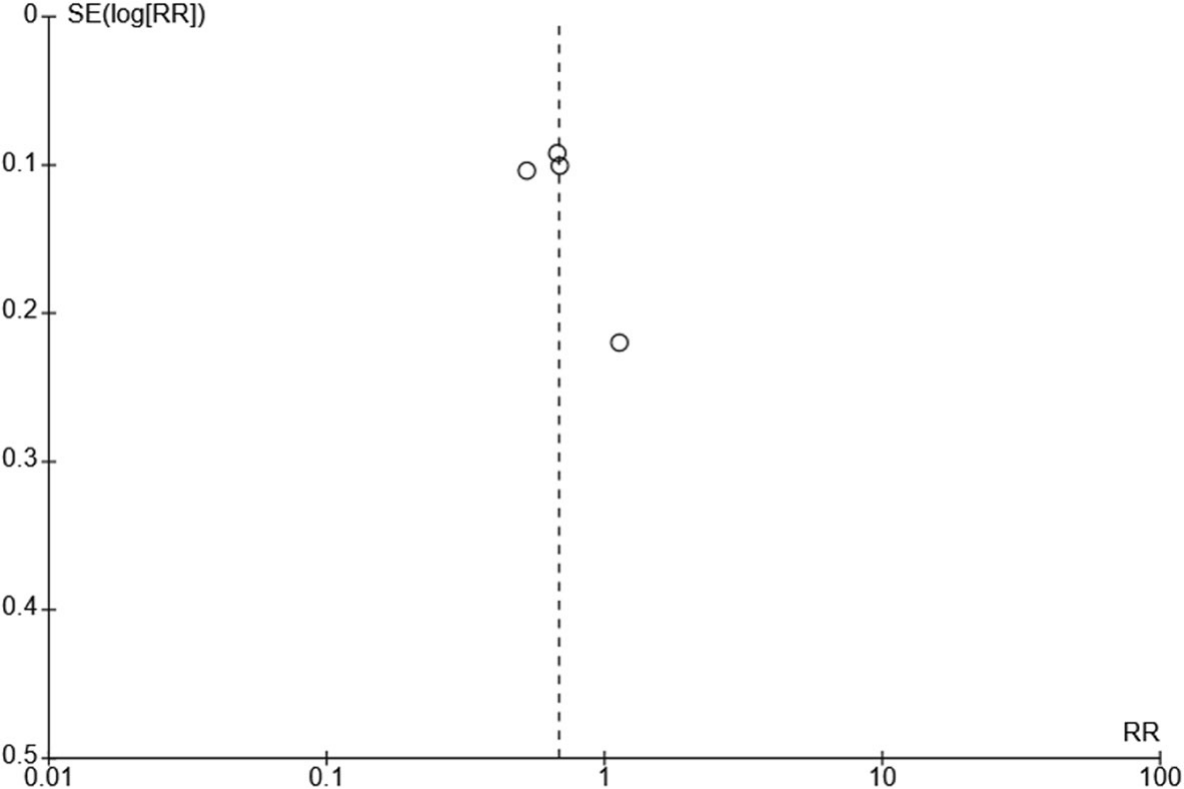
Funnel plot of comparison: NSAID vs antibiotic on Day 3 or 4 of symptomatic cure

Based on visual inspection, there was no asymmetry in the funnel plot, which suggests absence of publication bias, however, it still needs to be interpreted with caution (Fig. 8). Tests for funnel plot asymmetry to evaluate small-study effects, such as use of Egger and Begg’s test, were not applied since there were only four studies included in the meta-analysis, rendering low power of the tests. On assessment using the Cochrane risk of bias tool, there was no apparent selective non-reporting of results after comparing the reported outcomes and the plans that were pre-specified in the study protocol and in the methodological section of the actual study.

### Certainty of evidence

Using the GRADE approach (Table 2), three important outcomes, which comprise of symptom resolution by Day 3 or 4 of treatment, upper urinary tract infection complication and positive urine culture on follow-up, had moderate certainty of evidence. For the outcome use of secondary or rescue antibiotic due to persistent or worsening symptoms of UTI, the final certainty of evidence was graded as low. Primary reasons identified in this study for rating down the certainty of evidence per outcome included concerns for bias that could likely lower the confidence in the estimate of effect or due to inconsistency of results or if both, could lead to severe downgrade of evidence level.

## Discussion

### Summary of key findings and comparison with literature

This systematic review evaluated evidence from four European double-blinded randomized control trials involving 1165 patients with uncomplicated urinary tract infection conducted between 2007 to 2016.

Consolidating all the findings above, this meta-analysis determined that the treatment of uncomplicated UTI with non-steroidal anti-inflammatory drugs does not substantially improve symptom burden and achieve symptomatic cure, compounded further by complication risks as compared to standard antibiotic use. Our sensitivity analyses showed that the effectiveness of symptom resolution is less with NSAID treatment than use of antibiotics (RR: 0.63, 95% CIs [0.53, 0.74], *p* < 0.0001, I^2^ = 56%, moderate certainty of evidence). Moreover, the pooled number needed to harm is 4.76 for persistence of symptoms on Day 3 or 4 of illness (NNH 4.76, 95% CIs [12.5, 2.94]), with a risk difference of 0.21 (RD 0.21, 95% CIs [0.08, 0.34], I^2^ = 78%) (Fig. 3B).

The initial hypothesis of the possible use of NSAIDs in symptomatic treatment of uncomplicated urinary tract infections stems from the postulate that inflammation plays a key role in the development of lower urinary tract symptoms, with high sensitivity C-reactive protein as a marker of inflammation. Previous studies reported a significant association between CRP levels and lower urinary tract symptoms in women and men [21, 22], and subsequently among urinary tract infection patients [23]. Theoretically, NSAIDs may reduce contraction of the bladder muscle, which is partly responsible for the lower urinary tract symptoms, by inhibiting expression of cyclooxygenase 2, and synthesis of prostaglandins. This pathogenic mechanism was replicated in the study by *Takagi-Matsumoto* et al., which revealed dose-dependent suppression of rhythmic contraction induced by distension on normal and cystitis rats with intravenous administration of aspirin, indomethacin or ketoprofen [24]. However, it did not translate well in the clinical setting. In the 2019 ATAFUTI trial, there was no evidence of differences between those who took Ibuprofen advice vs. no advice in terms of symptom severity after 2–4 days of intervention (LS [least square] mean − 0.01, 95% CI [− 0.27, 0.26], *p* = 0.951) [17]. Also, in a randomized controlled pilot study by *Ko et. al.*, no significant difference was observed in the degree of pain scale reduction between those who took cepodoxime plus aceclofenac versus cepodoxime alone on Day 3 of treatment (*p* = 0.134) [20].

The non-steroidal anti-inflammatory drugs are known for its anti-inflammatory, analgesic and anti-pyretic properties. Nevertheless, some studies have demonstrated moderate to strong in vitro antimicrobial activity when tested against different bacterial isolates of Gram-positive and Gram-negative organisms. Aspirin inhibited all of the *S. aureus* and *E. faecalis* isolates of UTI at the most effective concentration of 500 μg/ml [25]. Conversely, diclofenac exhibited in vitro inhibition on 67% of clinically isolated strains of *E.coli*, with MIC values ranging from 5 to 50 μg/ml [26]. In case of Ibuprofen, zones of inhibition were observed for *S. aureus*, *B.subtilis*, *C. albicans* and *A. brasiliensis*, hence signifying a broad spectrum of activity for bacterial and fungal strains [27]. However, contrary to the previous studies, a recent investigation revealed that Ibuprofen lacks direct antimicrobial properties for treatment of urinary tract infection isolates without any effect on bacterial growth of *E. coli* or *E. faecalis* [28]. In our study, results revealed that the non-steroidal anti-inflammatory group is more likely to have a persistence of a positive microbiologic urine culture compared to use of antibiotics after treatment (RR: 2.77, 95% CIs [1.95, 3.94], *p* < 0.00001, I^2^ = 36%, moderate certainty of evidence). Failure to reach a bacteriologic cure can delay or not effectively attain optimal symptom improvement. This was shown in one of the reviewed studies by *Vik et. al.* as patients in the ibuprofen group who have a positive culture had a higher symptom burden and longer duration of lower urinary tract symptoms than those with a negative culture [15].

Another key finding in this study showed that while 43.34% of women in the combined trials treated with NSAIDs achieved symptomatic cure, the observed odds of developing upper UTI complications (pyelonephritis, and febrile UTI) was significantly higher in the NSAID group (Peto OR: 6.49, 95% CIs [3.02, 13.92], *p* < 0.00001, I^2^ = 0%, moderate certainty of evidence). This was due to the delay in instituting antibiotics. Due to the persistence or worsening of symptoms, patients who were given NSAID initially needed secondary treatment with antibiotics (RR: 3.16, 95% CIs [2.24, 4.44], *p* < 0.00001, I^2^ = 47%, low certainty of evidence). In the study by *Vik et. al*., some even required hospitalization for IV antibiotics (2 out of 7 with serious adverse events) [15]. While it is an important outcome that at least 40% of patients were spared of antibiotics, we cannot disregard those who were harmed during the process.

These results suggest that NSAIDS are not comparable to antibiotic treatment for uncomplicated UTI. Giving NSAIDs can reduce the use of antibiotics [13], but at the expense of high symptom burden, longer duration of symptoms, and more cases of progression to pyelonephritis.

Previous studies have compared the use of placebo and antibiotics in uncomplicated UTI favoring the administration of antibiotics due to the worsening of complications in the other placebo arm [5]. The same conclusion can be made in this study.

### Strengths and limitations

This manuscript presented a comprehensive systematic review and meta-analyses of non-steroidal anti-inflammatory drugs versus antibiotics for treatment of uncomplicated UTI with analyses of outcomes not only on efficacy but also on safety. A systematic critical appraisal of included studies was done using the updated Cochrane Risk of Bias 2.0 tool, and application of GRADE framework [29] to determine certainty of evidence. However, despite an in-depth search, this systematic review was limited to a paucity of randomized controlled trials available for this meta-analysis. There was also substantial heterogeneity among studies with observed variability in clinical characteristics, methodological design plus quality, and statistically. Other potential sources of bias in each study remain to be elucidated which may affect the magnitude of effect estimate.

### Implications for practice and research

In practice, aside from examining the overall effect estimate in an outcome, the overall certainty of evidence must also be considered to assist physicians in the clinical decision-making process together with the patients.

Future researches may need for more studies with improved methodological designs and execution, with an emphasis on appropriate use of statistical analyses, likewise stating explicit reasons for the missing outcome data and how it was addressed to correct for bias, and also ensure that pre-specified plans are concurrent with those presented in the published report. Due to some unexplained inconsistencies or heterogeneities in the study results, one may implement an individual participant data meta-analysis, or do more studies in relevant subgroups.

## Conclusion

This meta-analysis showed that antibiotic treatment was more effective than use of non-steroidal anti-inflammatory drugs for acute uncomplicated lower urinary tract infection with an overall moderate certainty of evidence. We recommend maintaining the use of empiric antibiotic therapy as the primary treatment for uncomplicated urinary tract infection because of limited non-steroidal anti-inflammatory drug benefits in symptom resolution, compounded by complication risks of developing upper UTI, with presence of positive urine culture even after treatment, and the need to use a secondary or rescue antibiotic. Despite the limited benefits of NSAIDs, there are at least a subgroup of patients who are responsive with spontaneous resolution of symptoms without a need for antibiotics. Future studies should emphasize on identifying aspects of underlying predictors and moderators of treatment effects.

## Supplementary Information

**Additional file 1.**

## Data Availability

The datasets used and/or analysed during the current study are available from the corresponding author on reasonable request.

## Abbreviations

NSAIDs: Non-steroidal anti-inflammatory drugs
UTI: Urinary tract infections
