# Supplementary material for: Symptomatic treatment (using NSAIDS) versus antibiotics in uncomplicated lower urinary tract infection: a meta-analysis and systematic review of randomized controlled trials
Source: BMC Infect Dis. 2021 Jun 29;21:619. doi: 10.1186/s12879-021-06323-0 (PMC8243445; doi:10.1186/s12879-021-06323-0)
Supplement: Supplementary file 1 — Additional file 1. [file 12879_2021_6323_MOESM1_ESM.docx]

**ASSESSING FOR RISK OF BIAS USING ROB 2 Tool**

Response options: Y / PY / PN / N / NI

Risk-of-bias judgment: Low / High / Some concerns

**OUTCOME: Symptom resolution by Day 3 or 4**

1. **Risk of Bias Arising from Randomization Process**

| **Signalling Question** | **Bleidorn** | **Gagyor** | **Kronenberg** | **Vik** |
| --- | --- | --- | --- | --- |
| **1.1 Was the allocation sequence random?** | Y | Y | Y | Y |
| **1.2 Was the allocation sequence concealed until** **participants were enrolled and assigned to intervention?** | Y | Y | Y | Y |
| **1.3 Did baseline differences between intervention groups suggest a problem with the randomization process?** | N | PN | N | N |
| **Risk of Bias Judgement** | **Low** | **Low** | **Low** | **Low** |

- 1. **Risk of Bias due to deviations from the intended interventions (effect of assignment to intervention)**

| **Signalling Question** | **Bleidorn** | **Gagyor** | **Kronenberg** | **Vik** |
| --- | --- | --- | --- | --- |
| **2.1 Were participants aware of their assigned intervention during the trial?** | N | N | N | N |
| **2.2 Were carers and people delivering the interventions aware of participant’s assigned intervention during the trial?** | N | N | N | N |
| **2.3 If Y/PY/NI to 2.1 or 2.2: Were there deviations from the intended intervention that arose because of the trial context?** |  |  |  |  |
| **2.4 If Y/PY/NI to 2.3: Were these deviations likely to have affected the outcome?** |  |  |  |  |
| **2.5 If Y/PY to 2.4: Were these deviations from intended intervention balanced between groups?** |  |  |  |  |
| **2.6 Was an appropriate analysis used to estimate the effect of assignment to intervention?** | PN | PN | Y | PY |
| **2.7 If N/PN to 2.6: Was there potential for a substantial impact (on the result) of the failure to analyse participants in the group to which they were randomized?** | PN | PN |  |  |
| **Risk of Bias Judgement** | **Some concerns** | **Some concerns** | **Low** | **Low** |

1. **Risk of bias due to missing outcome data**

| **Signalling Question** | **Bleidorn** | **Gagyor** | **Kronenberg** | **Vik** |
| --- | --- | --- | --- | --- |
| **3.1 Were data for this outcome available for all, or nearly all, randomized?** | N | N | N | Y |
| **3.2 If N/PN/NI to 3.1: Is there evidence that the result was not biased by missing outcome data?** | PN | PN | PN |  |
| **3.3 If N/PN/NI to 3.2: Could missingness in the outcome depend on its true value?** | PN | NI | PN |  |
| **3.4 If Y/PY/NI to 3.3: Is it likely that missingness in the outcome depended on its true value?** |  | PN |  |  |
| **Risk of Bias judgement** | **Low** | **Some concerns** | **Low** | **Low** |

1. **Risk of bias in measurement of the outcome**

| **Signalling Question** | **Bleidorn** | **Gagyor** | **Kronenberg** | **Vik** |
| --- | --- | --- | --- | --- |
| **4.1 Was the method of measuring the outcome inappropriate?** | N | N | N | N |
| **4.2 Could measurement or ascertainment have differed between intervention groups?** | N | N | N | N |
| **4.3 If N/PN/NI to 4.1 and 4.2: Were outcome assessors aware of the intervention received by study participants?** | N | N | N | N |
| **4.4 If Y/PY/NI to 4.3: Could assessment of the outcome have been influenced by knowledge of intervention received?** |  |  |  |  |
| **4.5 If Y/PY/NI to 4.4: Is it likely that assessment of the outcome was influenced by knowledge of intervention received?** |  |  |  |  |
| **Risk of Bias Judgement** | **Low** | **Low** | **Low** | **Low** |

1. **Risk of bias in selection of the reported result**

| **Signalling Question** | **Bleidorn** | **Gagyor** | **Kronenberg** | **Vik** |
| --- | --- | --- | --- | --- |
| **5.1 Were the data that produced this result analysed in accordance with a pre-specified analysis plan that was finalized before unblinded outcome data were available for analysis?** | N | Y | Y | Y |
| **Is the numerical result being assessed likely to have been selected on the basis of the results, from…**  **5.2…multiple eligible outcome measurements (e.g scales, definitions, time points) within the outcome domain?** | N | N | N | N |
| **5.3…multiple eligible analyses of the data?** | N | N | N | N |
| **Risk of Bias Judgement** | **Some concerns** | **Low** | **Low** | **Low** |

1. **Overall Risk of Bias**

|  | **Bleidorn** | **Gagyor** | **Kronenberg** | **Vik** |
| --- | --- | --- | --- | --- |
| **Randomization Process** | Low | Low | Low | Low |
| **Assignment to Intervention** | Some concerns | Some concerns | Low | Low |
| **Missing Outcome Data** | Low | Some concerns | Low | Low |
| **Measurement of Outcome** | Low | Low | Low | Low |
| **Selection of Reported Result** | Some concerns | Low | Low | Low |
| **Overall Risk** | **Some concerns** | **Some concerns** | **Low** | **Low** |

**OUTCOME: Complication of upper urinary tract infection (pyelonephritis/febrile UTI)**

1. **Risk of Bias Arising from Randomization Process**

| **Signalling Question** | **Gagyor** | **Kronenberg** | **Vik** |
| --- | --- | --- | --- |
| **1.1 Was the allocation sequence random?** | Y | Y | Y |
| **1.2 Was the allocation sequence concealed until** **participants were enrolled and assigned to intervention?** | Y | Y | Y |
| **1.3 Did baseline differences between intervention groups suggest a problem with the randomization process?** | PN | N | N |
| **Risk of Bias Judgement** | **Low** | **Low** | **Low** |

1. **Risk of Bias due to deviations from the intended interventions (effect of assignment to intervention)**

| **Signalling Question** | **Gagyor** | **Kronenberg** | **Vik** |
| --- | --- | --- | --- |
| **2.1 Were participants aware of their assigned intervention during the trial?** | N | N | N |
| **2.2 Were carers and people delivering the interventions aware of participant’s assigned intervention during the trial?** | N | N | N |
| **2.3 If Y/PY/NI to 2.1 or 2.2: Were there deviations from the intended intervention that arose because of the trial context?** |  |  |  |
| **2.4 If Y/PY/NI to 2.3: Were these deviations likely to have affected the outcome?** |  |  |  |
| **2.5 If Y/PY to 2.4: Were these deviations from intended intervention balanced between groups?** |  |  |  |
| **2.6 Was an appropriate analysis used to estimate the effect of assignment to intervention?** | Y | Y | PN |
| **2.7 If N/PN to 2.6: Was there potential for a substantial impact (on the result) of the failure to analyse participants in the group to which they were randomized?** |  |  | N |
| **Risk of Bias Judgement** | **Low** | **Low** | **Some concerns** |

1. **Risk of bias due to missing outcome data**

| **Signalling Question** | **Gagyor** | **Kronenberg** | **Vik** |
| --- | --- | --- | --- |
| **3.1 Were data for this outcome available for all, or nearly all, randomized?** | N | PY | N |
| **3.2 If N/PN/NI to 3.1: Is there evidence that the result was not biased by missing outcome data?** | PN |  | N |
| **3.3 If N/PN/NI to 3.2: Could missingness in the outcome depend on its true value?** | PN |  | PY |
| **3.4 If Y/PY/NI to 3.3: Is it likely that missingness in the outcome depended on its true value?** |  |  | PY |
| **Risk of Bias judgement** | **Low** | **Low** | **High risk** |

1. **Risk of bias in measurement of the outcome**

| **Signalling Question** | **Gagyor** | **Kronenberg** | **Vik** |
| --- | --- | --- | --- |
| **4.1 Was the method of measuring the outcome inappropriate?** | N | N | N |
| **4.2 Could measurement or ascertainment have differed between intervention groups?** | N | N | N |
| **4.3 If N/PN/NI to 4.1 and 4.2: Were outcome assessors aware of the intervention received by study participants?** | N | N | N |
| **4.4 If Y/PY/NI to 4.3: Could assessment of the outcome have been influenced by knowledge of intervention received?** |  |  |  |
| **4.5 If Y/PY/NI to 4.4: Is it likely that assessment of the outcome was influenced by knowledge of intervention received?** |  |  |  |
| **Risk of Bias Judgement** | **Low** | **Low** | **Low** |

1. **Risk of bias in selection of the reported result**

| **Signalling Question** | **Gagyor** | **Kronenberg** | **Vik** |
| --- | --- | --- | --- |
| **5.1 Were the data that produced this result analysed in accordance with a pre-specified analysis plan that was finalized before unblinded outcome data were available for analysis?** | Y | Y | PN |
| **Is the numerical result being assessed likely to have been selected on the basis of the results, from…**  **5.2…multiple eligible outcome measurements (e.g scales, definitions, time points) within the outcome domain?** | N | N | N |
| **5.3…multiple eligible analyses of the data?** | N | N | N |
| **Risk of Bias Judgement** | **Low** | **Low** | **Some concerns** |

1. **Overall Risk of Bias**

|  | **Gagyor** | **Kronenberg** | **Vik** |
| --- | --- | --- | --- |
| **Randomization Process** | Low | Low | Low |
| **Assignment to Intervention** | Low | Low | Some concerns |
| **Missing Outcome Data** | Low | Low | High risk |
| **Measurement of Outcome** | Low | Low | Low |
| **Selection of Reported Result** | Low | Low | Some concerns |
| **Overall Risk** | **Low** | **Low** | **High risk** |

**OUTCOME: Positive urine culture post-treatment**

1. **Risk of Bias Arising from Randomization Process**

| **Signalling Question** | **Bleidorn** | **Kronenberg** | **Vik** |
| --- | --- | --- | --- |
| **1.1 Was the allocation sequence random?** | Y | Y | Y |
| **1.2 Was the allocation sequence concealed until** **participants were enrolled and assigned to intervention?** | Y | Y | Y |
| **1.3 Did baseline differences between intervention groups suggest a problem with the randomization process?** | N | N | N |
| **Risk of Bias Judgement** | **Low** | **Low** | **Low** |

1. **Risk of Bias due to deviations from the intended interventions (effect of assignment to intervention)**

| **Signalling Question** | **Bleidorn** | **Kronenberg** | **Vik** |
| --- | --- | --- | --- |
| **2.1 Were participants aware of their assigned intervention during the trial?** | N | N | N |
| **2.2 Were carers and people delivering the interventions aware of participant’s assigned intervention during the trial?** | N | N | N |
| **2.3 If Y/PY/NI to 2.1 or 2.2: Were there deviations from the intended intervention that arose because of the trial context?** |  |  |  |
| **2.4 If Y/PY/NI to 2.3: Were these deviations likely to have affected the outcome?** |  |  |  |
| **2.5 If Y/PY to 2.4: Were these deviations from intended intervention balanced between groups?** |  |  |  |
| **2.6 Was an appropriate analysis used to estimate the effect of assignment to intervention?** | N | Y | N |
| **2.7 If N/PN to 2.6: Was there potential for a substantial impact (on the result) of the failure to analyse participants in the group to which they were randomized?** | PN |  |  |
| **Risk of Bias Judgement** | **Some concerns** | **Low** | **Some concerns** |

1. **Risk of bias due to missing outcome data**

| **Signalling Question** | **Bleidorn** | **Kronenberg** | **Vik** |
| --- | --- | --- | --- |
| **3.1 Were data for this outcome available for all, or nearly all, randomized?** | N | PY | N |
| **3.2 If N/PN/NI to 3.1: Is there evidence that the result was not biased by missing outcome data?** | PN |  | PN |
| **3.3 If N/PN/NI to 3.2: Could missingness in the outcome depend on its true value?** | N |  | PY |
| **3.4 If Y/PY/NI to 3.3: Is it likely that missingness in the outcome depended on its true value?** |  |  | N |
| **Risk of Bias judgement** | **Low** | **Low** | **Some concerns** |

1. **Risk of bias in measurement of the outcome**

| **Signalling Question** | **Bleidorn** | **Kronenberg** | **Vik** |
| --- | --- | --- | --- |
| **4.1 Was the method of measuring the outcome inappropriate?** | Y | N | Y |
| **4.2 Could measurement or ascertainment have differed between intervention groups?** | N | N | N |
| **4.3 If N/PN/NI to 4.1 and 4.2: Were outcome assessors aware of the intervention received by study participants?** | NI | N | NI |
| **4.4 If Y/PY/NI to 4.3: Could assessment of the outcome have been influenced by knowledge of intervention received?** | N |  | N |
| **4.5 If Y/PY/NI to 4.4: Is it likely that assessment of the outcome was influenced by knowledge of intervention received?** |  |  |  |
| **Risk of Bias Judgement** | **Low** | **Low** | **Low** |

1. **Risk of bias in selection of the reported result**

| **Signalling Question** | **Bleidorn** | **Kronenberg** | **Vik** |
| --- | --- | --- | --- |
| **5.1 Were the data that produced this result analysed in accordance with a pre-specified analysis plan that was finalized before unblinded outcome data were available for analysis?** | N | Y | Y |
| **Is the numerical result being assessed likely to have been selected on the basis of the results, from…**  **5.2…multiple eligible outcome measurements (e.g scales, definitions, time points) within the outcome domain?** | N | N | N |
| **5.3…multiple eligible analyses of the data?** | N | N | N |
| **Risk of Bias Judgement** | **Some concerns** | **Low** | **Low** |

1. **Overall Risk of Bias**

|  | **Bleidorn** | **Kronenberg** | **Vik** |
| --- | --- | --- | --- |
| **Randomization Process** | Low | Low | Low |
| **Assignment to Intervention** | Some concerns | Low | Some concerns |
| **Missing Outcome Data** | Low | Low | Some concerns |
| **Measurement of Outcome** | Low | Low | Low |
| **Selection of Reported Result** | Some concerns | Low | Low |
| **Overall Risk** | **Some concerns** | **Low** | **Some concerns** |

**OUTCOME: Secondary or rescue antibiotic**

1. **Risk of Bias Arising from Randomization Process**

| **Signalling Question** | **Bleidorn** | **Gagyor** | **Kronenberg** | **Vik** |
| --- | --- | --- | --- | --- |
| **1.1 Was the allocation sequence random?** | Y | Y | Y | Y |
| **1.2 Was the allocation sequence concealed until** **participants were enrolled and assigned to intervention?** | Y | Y | Y | Y |
| **1.3 Did baseline differences between intervention groups suggest a problem with the randomization process?** | N | PN | N | N |
| **Risk of Bias Judgement** | **Low** | **Low** | **Low** | **Low** |

1. **Risk of Bias due to deviations from the intended interventions (effect of assignment to intervention)**

| **Signalling Question** | **Bleidorn** | **Gagyor** | **Kronenberg** | **Vik** |
| --- | --- | --- | --- | --- |
| **2.1 Were participants aware of their assigned intervention during the trial?** | N | N | N | N |
| **2.2 Were carers and people delivering the interventions aware of participant’s assigned intervention during the trial?** | N | N | N | N |
| **2.3 If Y/PY/NI to 2.1 or 2.2: Were there deviations from the intended intervention that arose because of the trial context?** |  |  |  |  |
| **2.4 If Y/PY/NI to 2.3: Were these deviations likely to have affected the outcome?** |  |  |  |  |
| **2.5 If Y/PY to 2.4: Were these deviations from intended intervention balanced between groups?** |  |  |  |  |
| **2.6 Was an appropriate analysis used to estimate the effect of assignment to intervention?** | PN | Y | Y | PN |
| **2.7 If N/PN to 2.6: Was there potential for a substantial impact (on the result) of the failure to analyse participants in the group to which they were randomized?** | PN |  |  |  |
| **Risk of Bias Judgement** | **Some concerns** | **Low** | **Low** | **Some concerns** |

1. **Risk of bias due to missing outcome data**

| **Signalling Question** | **Bleidorn** | **Gagyor** | **Kronenberg** | **Vik** |
| --- | --- | --- | --- | --- |
| **3.1 Were data for this outcome available for all, or nearly all, randomized?** | N | N | N | N |
| **3.2 If N/PN/NI to 3.1: Is there evidence that the result was not biased by missing outcome data?** | PN | PN | PN | N |
| **3.3 If N/PN/NI to 3.2: Could missingness in the outcome depend on its true value?** | N | NI | N | PY |
| **3.4 If Y/PY/NI to 3.3: Is it likely that missingness in the outcome depended on its true value?** |  | PN |  | PY |
| **Risk of Bias judgement** | **Low** | **Some concerns** | **Low** | **High** |

1. **Risk of bias in measurement of the outcome**

| **Signalling Question** | **Bleidorn** | **Gagyor** | **Kronenberg** | **Vik** |
| --- | --- | --- | --- | --- |
| **4.1 Was the method of measuring the outcome inappropriate?** | N | N | N | N |
| **4.2 Could measurement or ascertainment have differed between intervention groups?** | N | N | N | N |
| **4.3 If N/PN/NI to 4.1 and 4.2: Were outcome assessors aware of the intervention received by study participants?** | N | N | N | N |
| **4.4 If Y/PY/NI to 4.3: Could assessment of the outcome have been influenced by knowledge of intervention received?** |  |  |  |  |
| **4.5 If Y/PY/NI to 4.4: Is it likely that assessment of the outcome was influenced by knowledge of intervention received?** |  |  |  |  |
| **Risk of Bias Judgement** | **Low** | **Low** | **Low** | **Low** |

1. **Risk of bias in selection of the reported result**

| **Signalling Question** | **Bleidorn** | **Gagyor** | **Kronenberg** | **Vik** |
| --- | --- | --- | --- | --- |
| **5.1 Were the data that produced this result analysed in accordance with a pre-specified analysis plan that was finalized before unblinded outcome data were available for analysis?** | N | Y | N | N |
| **Is the numerical result being assessed likely to have been selected on the basis of the results, from…**  **5.2…multiple eligible outcome measurements (e.g scales, definitions, time points) within the outcome domain?** | N | N | N | N |
| **5.3…multiple eligible analyses of the data?** | N | N | N | N |
| **Risk of Bias Judgement** | **Some concerns** | **Low** | **Some concerns** | **Some concerns** |

1. **Overall Risk of Bias**

|  | **Bleidorn** | **Gagyor** | **Kronenberg** | **Vik** |
| --- | --- | --- | --- | --- |
| **Randomization Process** | Low | Low | Low | Low |
| **Assignment to Intervention** | Some concerns | Low | Low | Some concerns |
| **Missing Outcome Data** | Low | Some concerns | Low | High risk |
| **Measurement of Outcome** | Low | Low | Low | Low |
| **Selection of Reported Result** | Some concerns | Low | Some concerns | Some concerns |
| **Overall Risk** | **Some concerns** | **Some concerns** | **Some concerns** | **High risk** |
